# Laser light in the era of pulsed field ablation — still a competitor?

**DOI:** 10.1007/s10840-023-01664-z

**Published:** 2023-11-06

**Authors:** Christian-Hendrik Heeger, Roland Richard Tilz

**Affiliations:** 1 Department of Rhythmologie, University Hospital Schleswig-Holstein, University Heart Center Lübeck, Lübeck, Germany; 2 German Center for Cardiovascular Research (DZHK), Partner Site Hamburg/Kiel/Lübeck, Lübeck, Germany

## Commentary to the article *Acute and long-term results with the 3rd generation visually guided laser balloon ablation system for PV isolation*

Cardiac catheter ablation procedures especially pulmonary vein isolation (PVI), as the currently most effective treatment option for atrial fibrillation (AF), are complex procedures with a relatively long learning curve. To reduce complexity and to improve safety, efficacy and efficiency single-shot devices have been introduced and shown promising acute and long-term success rates in numerous registries, studies, and randomized controlled clinical trials.1 Various catheter designs and energy sources have been evaluated in latest years, with the cryoballoon (CB) as the most common single-shot device with high level of clinical evidence [1]. Nevertheless, single-shot devices with a fixed size like the cryoballoon and the radiofrequency balloon have several limitations because the pulmonary vein anatomy strongly varies across patients [2, 3]. The visually guided laser balloon ablation system (HeartLight, CardioFocus, Marlborough, MA, USA) is a balloon-based ablation system which is utilizing laser light energy for lesion formation. Its design has been modified and optimized to its current version (X3, CardioFocus) and has been shown high PVI durability and similar clinical success compared to radiofrequency- and CB-based PVI [4, 5]. The X3 system offers a continuous sizeable balloon diameter and an automated continuous lesion formation [5].

In this issue of the *Journal of Interventional Cardiac Electrophysiology* Funasako et al. demonstrated safety, efficacy, and promising long-term follow-up utilizing the X3 for paroxysmal (PAF) and persistent AF (PersAF) patients. A total of 110 AF patients have been included and treated by the X3 laserballoon. The continuous ablation mode “RAPID mode” was applied to 99.5% of pulmonary veins, and 32.8% pulmonary veins were treated by the RAPID mode only. 91.1% of pulmonary veins were isolated on the first circumferential lesion and did not require any radiofrequency current touch up ablation. During the index procedure, 100% of pulmonary veins were successfully isolated. These observations connote that the X3 has been used as a real single-short device in about 1/3 of PVs with relative high rate of continuous lesion applications utilizing the RAPID mode. The mean procedure time was 77.0±22.7 min with a LA dwelling time of 61.9 ± 22.0 min. Periprocedural complications were limited to three transient phrenic nerve injuries. The 1-year AF free survival rate was 93.7% in PAF patients and 81.1% in PersAF patients.

Although these findings are promising there are several limitations utilizing the X3 system. Although a sizable balloon might lead to individualized PVI, this effect has not lead to improve outcome which was recently shown by Chun et al. comparing CB vs laser balloon-based PVI in AF patients [4]. Additionally the procedure time for X3 is longer in comparison to recent studies focusing on CB systems [4, 6]. As also observed in the studies by Funasako et al., Schmidt et al., and Heeger et al., a relatively high pinhole rate of 7–14%, leading to the necessity of a catheter exchange, has been reported for the X3 system [5, 7]. Due to these observations, the X3 balloon material has been recently modified to be more robust; however, there is no data available for this modification until today.

Contrary to thermal ablation energy sources like radiofrequency, laser light and cryotherapy pulsed field ablation (PFA) offers a unique non-thermal ablation modality with promising safety and efficacy advantages [8]. Cardiac tissue ablation is therefore not performed by thermal injury but by exposition to repetitive and rapid high-voltage electrical fields. Despite the fact that cardiac cells are destabilized within milliseconds, PFA offers the unique opportunity of selectivity to myocardial tissue while sparing non-myocardial cells. The combination of these two features (speed and selectivity) of PFA implies this novel ablation modality to be an ideal technology for safe, simple, fast, and effective single-shot devices which has been recently shown with the introduction of the FARAPULSE system. With this system, safe and effective PVI has been performed within a mean procedure time of 38±13 min in the 5S study and after further simplification and limitation to a single-sheath approach within 27.4±6.6 min [9, 10].

In summary, PVI by the X3 laser balloon system was safe, feasible, and effective in this single-center study. However, due to the fact that PFA offers fast, simple, safe, and selective cardiac tissue ablation, this novel modality might be the optimal energy source for single-shot devices.

Additionally, the current available PFA systems are only first generation, and further optimized PFA systems, catheter designs, and single-tip PFA catheter are approaching. Therefore, thermal energy modalities like laser light ablation procedures in its current form and catheter designs will have a hard times asserting themselves. However, as usual, only randomized clinical trials will offer powered data to draw final conclusions.
